# Castor stent reconstruction of left subclavian artery reduces the incidence of postoperative cerebral infarction of type B aortic dissection with insufficient proximal landing area: a propensity score matched analysis

**DOI:** 10.3389/fcvm.2026.1777371

**Published:** 2026-03-12

**Authors:** Ning Li, Shenglin Ge, Jianhao Hu, Bing Liu

**Affiliations:** 1 First Affiliated Hospital of Anhui Medical University, Hefei, China; 2 Hefei BOE Hospital, Hefei, China

**Keywords:** castor single-branched stent-graft, left subclavian artery, proximal anchoring, surgical procedures, thoracic endovascular aortic repair

## Abstract

**Background:**

Compared with conventional treatment of type B aortic dissection (TBAD) with insufficient proximal anchoring by covering the left subclavian artery (LSA), reconstructing LSA with Castor branch stent is a promising strategy. Prior studies lacked comparisons with partial LSA coverage, so we used propensity score matching analysis (PSMA) to retrospectively evaluate both approaches.

**Methods and results:**

We studied 377 patients with thoracic aortic dissection (TBAD) who underwent thoracic endovascular aortic repair (TEVAR) at one center. Of these, 262 had partial LSA coverage, and 115 had LSA reconstruction with a Castor stent. Using PSMA, we created 92 matched pairs for analysis. Kaplan–Meier and Cox regression analyses were conducted to assess the primary outcome of any postoperative cerebral infarction, encompassing both symptomatic strokes and silent brain infarcts, with each component also evaluated as secondary outcomes. Symptomatic strokes were confirmed by neurological symptoms combined with magnetic resonance imaging (MRI) or computed tomography (CT) scans, while silent brain infarcts were detected through routine postoperative CT/MRI scans for all patients, and both outcomes were included in the primary composite outcome of postoperative cerebral infarction. Additionally, all-cause mortality and postoperative LSA occlusion were examined. The Castor group showed less renal insufficiency (18.7% vs. 38.3%, *P* < 0.01) and was younger (52 vs. 62 years, *P* < 0.01). Baseline parameters were balanced after PSMAIn the matched cohort, overall median follow-up was 26 months (IQR 16–38); 30 months (IQR 22–38) for the Castor group and 24 months (IQR 14–36) for the partial coverage group, with total follow-up of 433.8 person-years (232.9 vs. 200.9 person-years). No notable differences existed in the unmatched cohort. Post-PSMA, the Castor group had significantly lower risk of postoperative cerebral infarction (HR 0.228, 95% CI 0.063–0.820, *P* = 0.013) and symptomatic stroke (HR 0.102, 95% CI 0.013–0.817, *P* = 0.008), with no difference in silent brain infarcts (HR 0.584, 95% CI 0.097–3.508, *P* = 0.552).

**Conclusions:**

In TBAD with insufficient proximal anchoring, Castor branch stent reconstruction reduces cerebral infarction over a median follow-up of 26 (IQR 16–38) months (433.8 total person-years), compared to partial LSA coverage.

## Introduction

Stanford type B aortic dissection (TBAD) represents a prevalent clinical emergency characterized by a significant threat to life and health, accompanied by a relatively high mortality rate. Most cases of TBAD are characterized by an acute onset and rapid progression (1–3). At present, thoracic endovascular aortic repair (TEVAR) is the preferred treatment modality for complicated TBAD, owing to its reduced complication rates and lower associated mortality (4–6). Medical management—focused on aggressive blood pressure control (targeting systolic blood pressure 100–120 mmHg) and pain relief—remains the first-line treatment for uncomplicated TBAD (5). However, certain limitations exist. One such limitation is the requirement for an adequate proximal landing area, which should range from 15 to 20 mm, as noted by previous studies (7). Therefore, for patients with TBAD whose primary entry tear is adjacent to or directly involves the LSA—a clinical scenario that results in an insufficient proximal landing zone for TEVAR—the stent graft may need to be extended across the LSA to achieve an adequate proximal aortic seal and establish a secure anchoring zone for successful endovascular repair. Numerous studies have indicated that the coverage of the LSA is associated with an elevated risk of endoleak, stroke, spinal cord ischemia, and upper limb ischemia (8–12). In addition, the incidence of long-term stroke after surgery will also increase significantly (7–10). A growing body of contemporary research has consistently demonstrated that preserving LSA perfusion confers meaningful short-term clinical benefits—including a reduced incidence of postoperative cerebral infarction and upper extremity ischemia—while also yielding improved long-term outcomes such as enhanced survival, better functional recovery, a lower risk of late-onset stroke, and a reduced need for secondary interventional procedures (7, 12–15). Common methods for LSA reconstruction and protection in TEVAR encompass the debranching technique (LSA bypass or transposition), the parallel technique, fenestrated stent grafts (customized fenestration or *in situ* fenestration), branched stent grafts, scalloped grafts, and stent grafts with a free flow (uncovered) segment positioned over the LSA ostium (16, 17).

Among these, branched stent grafts are capable of effectively reconstructing the blood supply to the LSA while also providing an adequate anchoring length to seal the intimal tear. In recent years, this method has been extensively utilized in clinical practice, demonstrating notable efficacy and safety (16, 18). However, the procedural complexity remains a challenge, and its long-term effects continue to be a subject of debate. Furthermore, our center observed that during treatment, employing a straight tube-type coated stent for partial coverage of the LSA ostium (ranging from one-third to two-thirds coverage) not only maintains LSA blood supply but also effectively seals the intimal tear. This method is straightforward to implement and necessitates reduced surgical time. At present, there is a paucity of comparative studies addressing the prognostic outcomes of these two techniques. This clinical conundrum, often encountered in practical settings, continues to be a topic of discussion. To address this issue, we conducted a retrospective study aimed at evaluating and comparing the therapeutic outcomes of both interventions in patients with TBAD and compromised proximal anchoring zones.

## Methods

This study was a single-center retrospective analysis conducted in adherence to the principles outlined in the Declaration of Helsinki. It received approval from the Ethics Committee of The First Affiliated Hospital of Anhui Medical University in August 2025 (Approval Number: PJ2025-06-78). Due to the retrospective design of the study, the ethics committee previously mentioned granted a waiver for obtaining informed consent from the patients.

This study encompassed a cohort of 377 patients diagnosed with Thoracic Aortic Dissection (TBAD) who were treated at the Cardiovascular Center of our department between January 2020 and March 2024. Based on the treatment modality applied to the LSA, the patients were stratified into two groups: the Partial Coverage group (*n* = 262) and the Castor stent group (*n* = 115).

### Patient cohort

TBAD; Inclusion criteria: (1) Pre-operative aortic computed tomography angiography (CTA) was performed to clearly diagnose TBAD (19). (2) the distance from the dissection tear to the distal end of the LSA ostium was less than 1.5 cm (7). (3) The left carotid artery (LCA) or left vertebral artery was not involved in the dissection, and the distance between the LCA or the single left vertebral artery of the aortic arch and the proximal edge of the dissected aorta was greater than 1.5 cm. (4) Persistent chest pain, refractory hypertension, concurrent pleural effusion, or pericardial hemorrhage were identified in the patients. (5) Acute-phase aortic dissection; Exclusion criteria: (1) Stanford type A dissection; (2) Abnormal surgical access that precluded interventional surgical treatment; (3) Malignant tumor; (4) Severe heart, lung, or liver failure; (5) History of previous TEVAR surgery; (6) Recent cerebral infarction or ischemia of major organs such as the liver and kidneys; (7) Allergy to iodine contrast agents; (8) Congenital connective tissue diseases, such as Marfan syndrome; (9) Subacute and chronic aortic dissection. Emergency surgical treatment was performed in patients with signs of organ malperfusion or aortic intimal tear; additionally, emergency surgery was indicated for those without such signs but complicated with persistent chest pain, refractory hypertension, hemorrhagic pleural effusion or pericardial effusion, aortic diameter >40 mm, aortotomy on the lesser curvature, or false lumen diameter >22 mm (1).

### TEVAR procedure

All patients underwent surgery under general anesthesia in a hybrid operating room. For the partial LSA coverage group, first, a 6F sheath was inserted percutaneously into the left brachial artery, and a 5F ordinary pigtail catheter with a guide wire was guided through the left brachial artery to the left dynamic marker of the ascending aorta. A 5F angiographic pigtail catheter with a guide wire was inserted from the right open femoral artery access through the true lumen and guided to the ascending aorta, and angiography was performed to measure the thoracic aorta. Then, based on the pre-operative CTA and intraoperative angiography measurements, a super—stiff guide wire was exchanged inside the angiographic pigtail catheter, and a stent with a size 5%—15% larger was deployed over the stiff wire through the open femoral artery access. The selection of an appropriate oversized size depended on the aortic anatomy and the surgeon's preference. Due to the insufficient proximal landing area, the LSA of the patients in this group was partially covered. The proximal edge of the LSA ostium was located in real - time based on the 5F pigtail catheter in the left brachial artery, and the covered part of the LSA was 1/3–2/3 of its diameter. After that, angiography was performed again to assess distal perfusion and intimal leakage. The Castor single—branched stent—graft (MicroPort Medical, Shanghai, China) has been approved by the China Food and Drug Administration and is the first single—branched stent—graft in China (20, 21). The surgical method of the Castor single—branched stent group for LSA revascularization was different from that of the partial LSA coverage group. First, a 6F sheath was inserted percutaneously into the left brachial artery and a 5F sheath was inserted percutaneously into the left femoral artery. A 10F sheath was inserted into the right open femoral artery. A 5F single—curve catheter combined with a super—slippery guide wire was introduced from the 6F sheath of the left brachial artery through the true lumen into the 10F sheath on the right side and extended out. A 5F angiographic pigtail catheter with a guide wire was guided from the 10F sheath of the right femoral artery through the true lumen to the ascending aorta, and angiography was performed to measure the thoracic aorta. An ordinary pigtail catheter with a guide wire was guided through the true lumen from the left femoral artery to the ascending aorta. A super—stiff guide wire was exchanged inside the angiographic pigtail catheter of the right femoral artery. This stiff guide wire was fixed, and the angiographic pigtail catheter was withdrawn. Then, the traction wire at the end of the branched graft was inserted into the single—curve catheter and pulled out from the left brachial artery. The main body of the Castor single—branched stent—graft entered the thoracic aorta along the stiff guide wire. At the same time, the single—curve catheter was combined with the traction wire and moved simultaneously with the main body of the Castor single—branched stent—graft. The position of the stent was adjusted according to the marks. The position of the stent was confirmed by angiography with an ordinary pigtail catheter through the left femoral artery. Then, the outer sheath and the soft sheath were removed, and the branched graft was dragged into the LSA by pulling the traction wire. Finally, the trigger wire was pulled to deploy the main body of the Castor single—branched stent, and then the traction wire was retracted. All patients in both groups were given aspirin (100 mg/day) after TEVAR.

### Data interpretation

The primary outcome assessed was cerebral infarction, while the secondary outcomes encompassed all-cause mortality, paraplegia, and vascular complications. Kaplan–Meier analysis and Cox proportional hazards regression were employed to evaluate postoperative cerebral infarction, all-cause mortality, and postoperative occlusion of the LSA. The primary outcome was defined as the duration from surgical intervention to the initial occurrence of cerebral infarction. In instances where cerebral infarction did not occur by the study's conclusion, the data were treated as censored. The definition of cerebral infarction encompassed both symptomatic cerebral infarction and silent brain infarcts (SBI).

PartialCoverage was characterized as a technique in TEVAR, which entails employing a straight tube-type covered stent to occlude one-third to two-thirds of the LSA ostium. Kaplan–Meier event-free survival curves were described as the likelihood that study participants have not encountered the specified event by a particular time point. In this study, symptomatic cerebral infarction was characterized as an acute-onset, vasculogenic focal or diffuse neurological deficit persisting for more than 24 h or resulting in death, with confirmation via magnetic resonance imaging (MRI) or computed tomography (CT) scans. Infarcts were assessed by investigators blinded to the treatment allocation. Silent brain infarctions (SBI) were identified as round, ovoid, or wedge-shaped hypodense parenchymal lesions on non-contrast cranial CT, or as T1-hypointense/T2-FLAIR-hyperintense lesions on brain MRI, with a diameter of ≥3 mm. These lesions were localized to a specific cerebral vascular territory, distinctly demarcated from normal brain tissue, and exhibited no associated mass effect or midline shift. SBIs were detected in patients who had no definite history of stroke or transient ischemic attack (TIA), and who exhibited no clinical symptoms or signs of neurological deficit related to the identified lesions. Participants with SBI underwent routine cranial MRI/CT scans at predefined follow-up intervals, and all infarct lesions were adjudicated by investigators blinded to the patients' treatment allocation.

### Data collection and follow-up

Patient data were obtained from hospital medical records. Follow-up was conducted either through direct telephone questionnaires or in-person at the outpatient clinic of our Cardiac Center, achieving a follow-up rate of 99.4%.

### Statistical analysis

Variables that do not follow a normal distribution are reported as the median (M) along with the first (Q1) and third quartiles (Q3), and comparisons are conducted using Mann–Whitney *U* tests. Categorical data are presented as frequencies and percentages [*n* (%)] and are analyzed using chi-square (*χ*^2^) tests or Fisher's exact tests, as appropriate. Propensity score matching is employed to control for confounding variables(A 1:1 greedy nearest neighbor matching algorithm was employed, utilizing a caliper of 0.02 standard deviations of the logit of the propensity score, and was conducted without replacement). Balanced 92 pairs across groups based on clinical covariates (age, gender, aortic calcification, Coronary heart disease, hypertension, Diabetes mellitus, Uremia, personal history of cerebral infarction, Respiratory diseases, pleural effusion, the distance from the intimal tear to the LSA ostium, the preoperative true lumen diameter, the preoperative false lumen diameter). The study utilized Kaplan–Meier survival curves accompanied by log-rank tests for survival analysis. Cox proportional hazards models were applied to evaluate the risks associated with events. Post-matching analyses employed paired Wilcoxon tests for continuous variables and McNemar tests for categorical variables. Statistical significance was determined at a threshold of *p* < 0.05, using SPSS version 25 and R version 4.4.1.

## Results

### Position and diameter measurement in aortic dissection

Preoperative enhanced CT of the aorta (arterial phase scanning) was performed with the patient in the supine position.

The measurement was conducted on the cross-sectional plane adjacent to the site of the aortic dissection tear. The “electronic caliper” tool available on the CTA workstation was employed for this purpose, with the measurement unit consistently set to millimeters (mm). The maximum diameter of the true lumen was recorded, with careful orientation of the measurement line perpendicular to the long axis of the true lumen. For the false lumen without thrombosis, located on the same measurement plane as the true lumen, the measurement procedure was analogous to that of the true lumen. The maximum diameter of the false lumen was determined, ensuring that the measurement line remained perpendicular to the long axis of the false lumen.

### Baseline characteristics

A total of 377 patients diagnosed with Thoracic Aortic Dissection (TBAD) were included in the study cohort. The average age of the participants was 60 years, with an interquartile range (IQR) of 50–71.5 years, and there was a predominance of male participants, accounting for 78.2% of the sample. The prevalence of comorbidities and preoperative characteristics among the patients were as follows: aortic calcification was observed in 26.0% of patients, coronary heart disease in 22.0%, hypertension in 81.9%, diabetes mellitus in 1.9%, uremia in 24.7%, a personal history of cerebral infarction in 24.4%, respiratory diseases in 4.0%, and pleural effusion in 18.0%.Furthermore, the distance from the intimal tear to the ostium of the LSA measured 5 mm [interquartile range (IQR), 2–10 mm]. The preoperative diameter of the true lumen was 11 mm (IQR, 3–13 mm), while the preoperative diameter of the false lumen was 18 mm (IQR, 16–21 mm). Specifically, partial coverage of the LSA (PartialCoverage group) was performed in 262 patients, and revascularization of the LSA using Castor branched stents (Castor group) was conducted in 115 patients. Prior to the implementation of PSMA, the Castor group (*n* = 115) demonstrated a significantly lower prevalence of renal insufficiency (18.7% compared to 38.3%, *p* < 0.01) and a younger mean age (52 years compared to 62 years, *p* < 0.01) relative to the PartialCoverage group (*n* = 262). No significant differences were observed between the two groups concerning other baseline characteristics, such as gender, aortic calcification, and coronary heart disease (all *p* > 0.05). The characteristics of the patients are detailed in Table 1.

**Table 1:** Patient characteristics.

Before PSMA				After PSMA		
	Castor (*n* = 115)	PartialCoverage (*n* = 262)	*p*	Castor (*n* = 92)	PartialCoverage （*n* = 92)	*p*
Age, years	52 (43–65)	62 (54–72)	<0.001	55 (44.25–64.75)	59.5 (46–69)	0.269
Gender			0.174			0.473
Male	95 (82.6)	200 (76.3)		75 (81.52)	70 (76.09)	
Female	20（17.4%)	62 (23.7)		17 (18.48)	22 (23.91)	
Aortic calcification	26 (22.6)	72 (27.5)	0.321	20 (21.7)	29 (31.5)	0.188
Coronary heart disease	25 (21.7)	58 (22.1)	0.932	19 (20.7)	29 (31.5)	0.143
Hypertension	99 (86.1）	210 (80.2)	0.168	79 (85.9)	83 (90.2)	0.053
Diabetes mellitus	4 (3.5)	3 (1.1)	0.207	1 (1.1)	2 (2.2)	1.000
Uremia	44 (38.3)	49 (18.7)	<0.001	34 (37)	37 (40.2)	0.743
History of cerebral infarction	27 (23.5)	65 (24.8)	0.782	19 (20.7)	25 (27.2)	0.405
Respiratory diseases	5 (4.3)	10 (3.8)	0.781	2 (2.2)	5 (5.4)	0.453
Pleural effusion	23 (20)	42 (16)	0.348	15 (16.3)	17 (18.5)	0.845
Distance from the intimal tear to the LSA ostium, mm	5.5 (2–11)	5 (2–10)	0.428	5 (2–10.7)	8 (2–11)	0.172
Preoperative true lumen, mm	11 (9–13)	10.5 (9–12)	0.359	11 (9–13)	10 (9–12)	0.271
Preoperative false lumen, mm	19 (17–22)	18 (16–21)	0.177	19 (16.25–22)	20 (18–22)	0.448

Data are shown in *n* (%), median (interquartile range; 25th–75th percentiles), *p*-value of <0.05 was considered statistically significant.

PSMA, propensity score matching analysis.

### Direct comparison of the two surgical approaches

The average surgical duration and postoperative in-hospital stay were 85 min [interquartile range (IQR), 65–85] and 5 days (IQR, 3–7), respectively. perioperative adverse events and outcomes were as follows: pulmonary infection occurred in 19 patients (5.0%), left brachial artery aneurysm in 12 patients (3.2%), aortic rupture in 2 patients (0.53%), recurrent aortic dissection or aneurysm in 9 patients (2.4%), endoleak in 29 patients (7.7%), all-cause mortality in 38 patients (10.0%), cerebral infarction in 24 patients (6.4%), paraplegia in 3 patients (0.8%), and postoperative LSA occlusion in 9 patients (2.4%). The median follow-up period was 29 months (IQR, 20–44). Notably, there were no instances of puncture site infection, postoperative renal failure, or graft migration observed in any patient following surgery. Subsequent subgroup analysis revealed no significant differences in complication rates between the two groups (all *p* > 0.05).

In terms of perioperative outcomes, the PartialCoverage group demonstrated a significantly reduced surgical duration [75 min [IQR, 60–90] compared to 115 min [IQR, 100–150]; *p* < 0.01] and a shorter postoperative in-hospital stay [4 days [IQR, 3–7] vs. 5 days [IQR, 4–7]; *p* = 0.019] relative to the Castor group. There was no statistically significant difference in the median follow-up period between the two groups, with the PartialCoverage group at 28 months [IQR, 20–47] and the Castor group at 30 months [IQR, 22–38]; *p* = 0.751. Furthermore, no significant differences were identified between the groups regarding perioperative adverse events, including pulmonary infection, left brachial artery aneurysm, puncture site infection, postoperative renal failure, aortic rupture, recurrent aortic dissection or aneurysm, graft migration, endoleak, all-cause mortality, cerebral infarction, paraplegia, and postoperative LSA occlusion (all *p* > 0.05).

**Figure 1:**
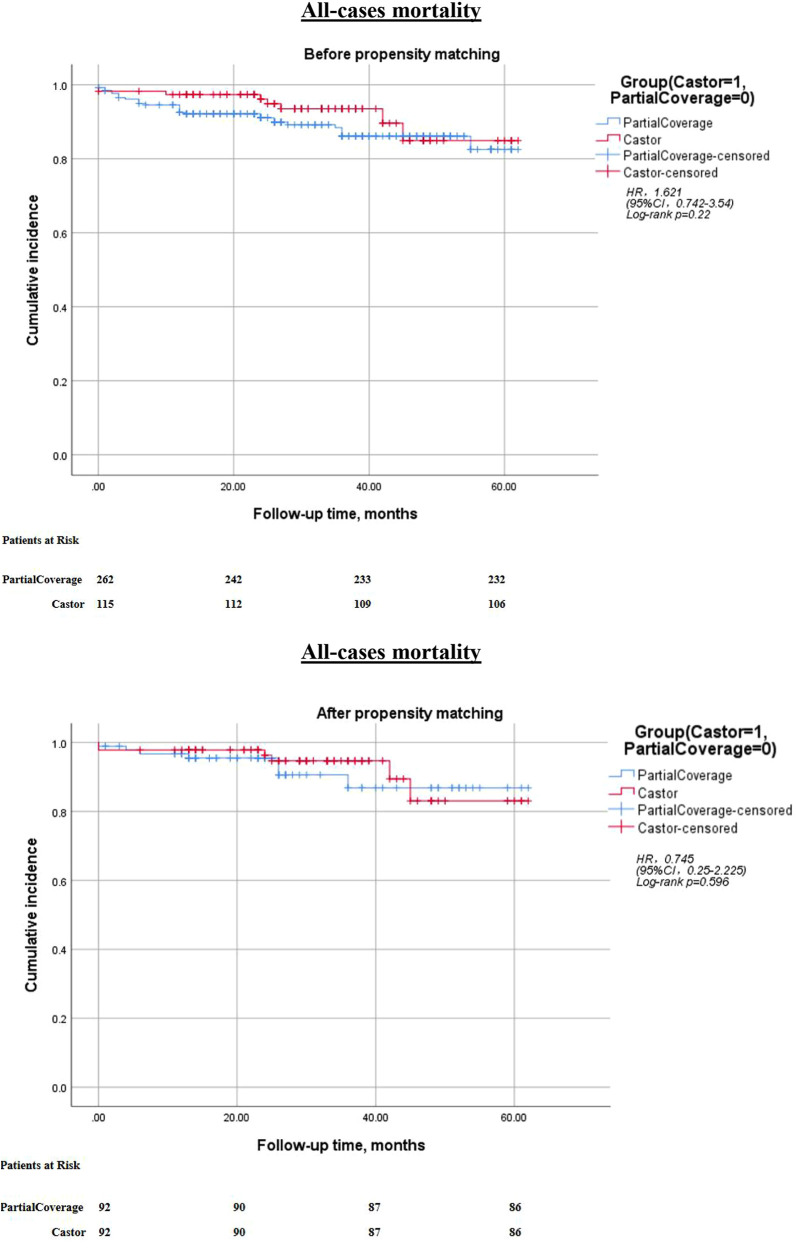
Kaplan–Meier cumulative event curves of all-cause mortality before and after prosperity score-matching. The Castor risk adjustment value relative to PartialCoverage is displayed. PartialCoverage: left subclavian artery (LSA) partial coverage surgery; Castor: Castor branch stent reconstruction left subclavian artery surgery; HR, hazard ratio; CI, confidence interval.

**Figure 2:**
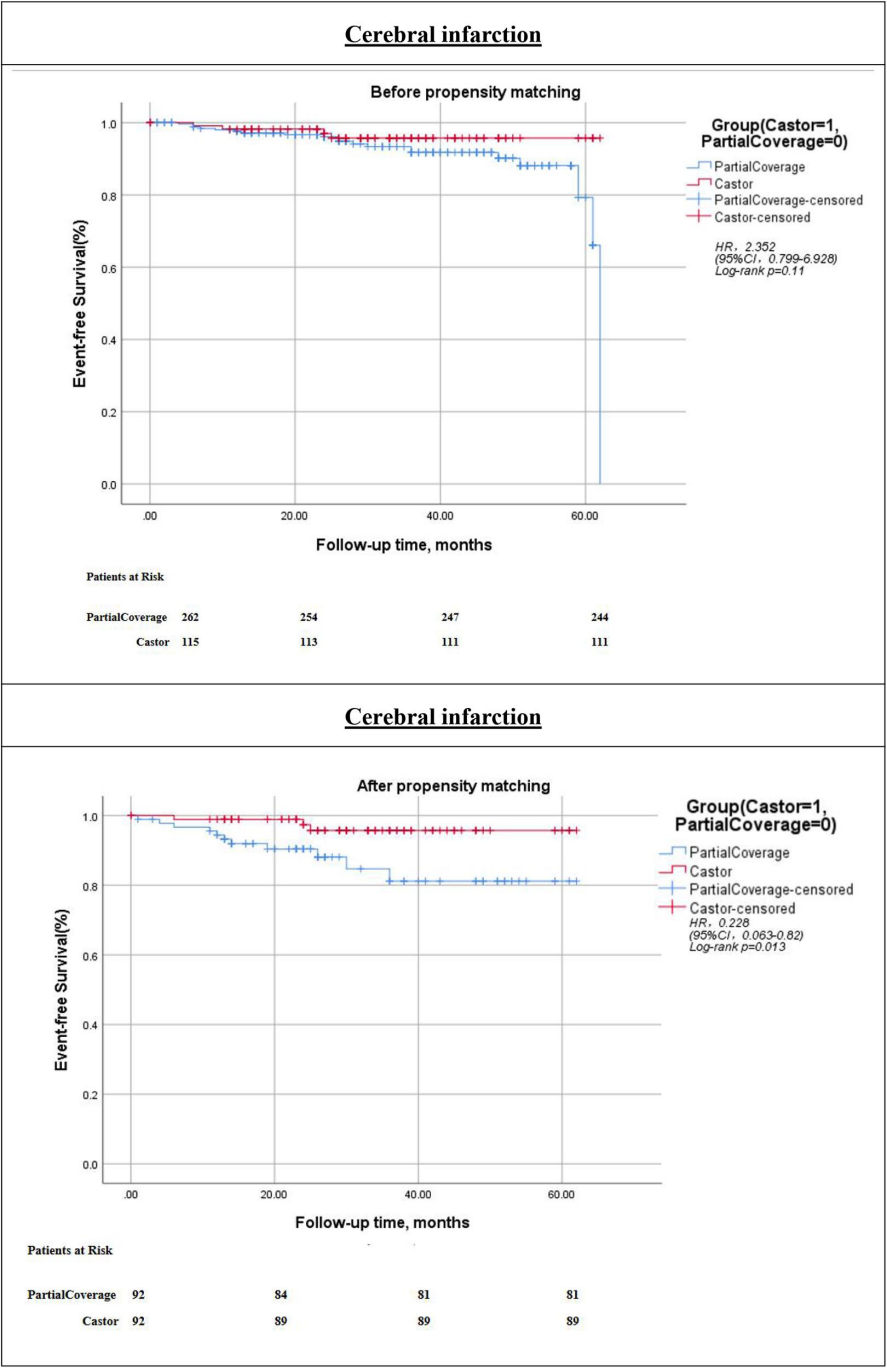
Kaplan–Meier cumulative event curves of cerebral infarction occurrence before and after prosperity score-matching. The Castor risk adjustment value relative to PartialCoverage is displayed. PartialCoverage: left subclavian artery (LSA) partial coverage surgery; Castor: Castor branch stent reconstruction left subclavian artery surgery; HR, hazard ratio; CI, confidence interval.

**Figure 3:**
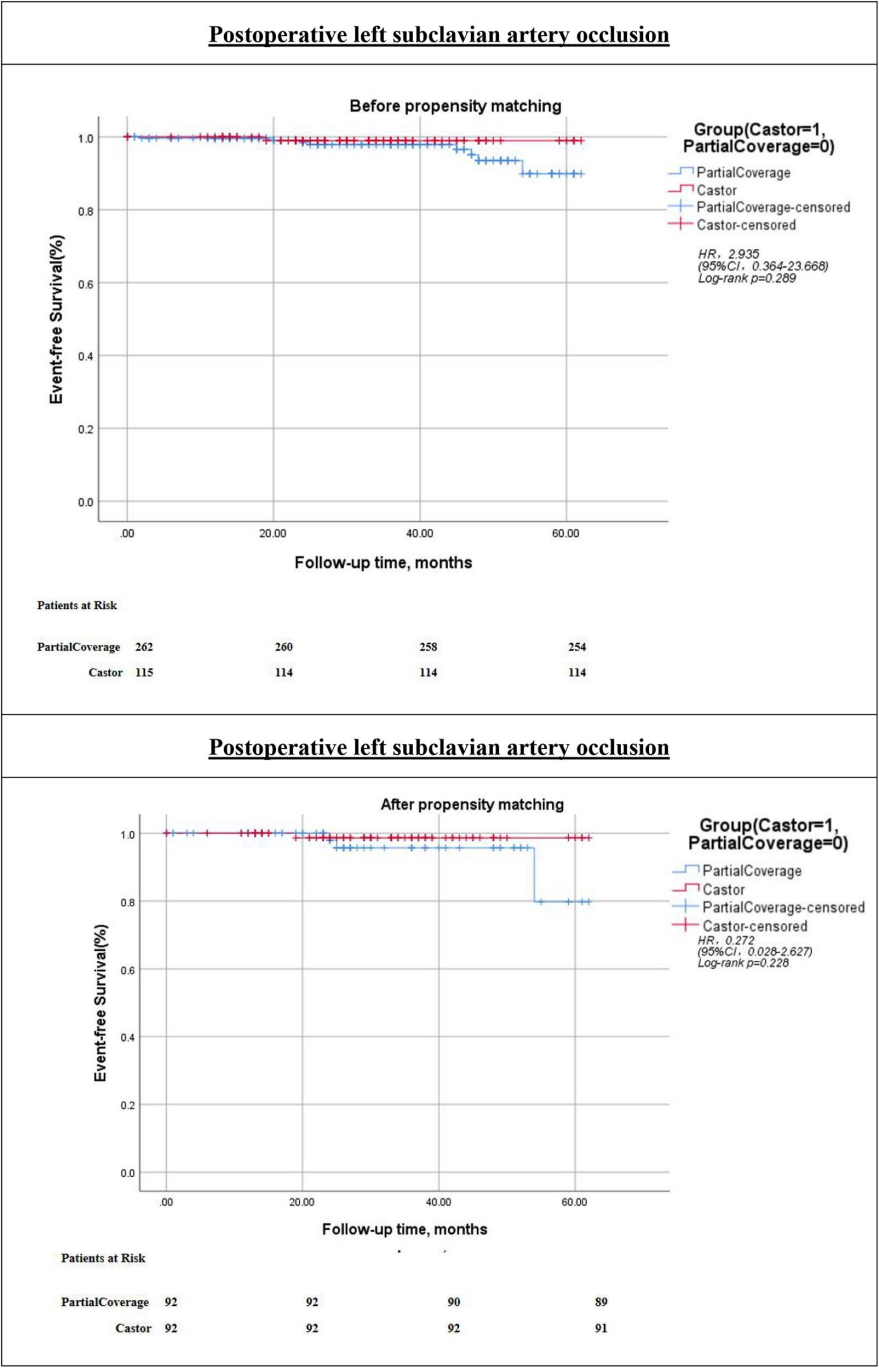
Kaplan–Meier event-free survival curves of postoperative left subclavian artery occurrence before and after prosperity score-matching. PartialCoverage: left subclavian artery (LSA) partial coverage surgery; Castor: Castor branch stent reconstruction left subclavian artery surgery; HR, hazard ratio; CI, confidence interval.

The technical success rate was 100% in both cohorts. Kaplan–Meier analysis and Cox regression modeling indicated that cerebral infarction-free survival did not differ significantly between the two groups [hazard ratio [HR] = 2.352; 95% confidence interval [CI], 0.799–6.928; *p* = 0.11]. Furthermore, there were no statistically significant differences between the groups concerning all-cause mortality or postoperative LSA occlusion-free survival (all *p* > 0.05) (Figures 1–3).

### PSMA-based comparison of the two surgical approaches

Initially, patients were categorized into two groups according to the surgical strategy employed. To enhance the comparability of preoperative characteristics between these groups, PSMA was conducted. This adjustment accounted for variables including age, gender, aortic calcification, coronary heart disease, hypertension, diabetes mellitus, uremia, personal history of cerebral infarction, respiratory diseases, pleural effusion, the distance from the intimal tear to the LSA ostium, preoperative true lumen diameter, and preoperative false lumen diameter. Following the matching process, a total of 184 patients were included, with 92 patients in each group, corresponding to the different surgical approaches. The baseline parameters were well-balanced between the two groups, as indicated by all *p*-values exceeding 0.05 (see Table 1). In the matched cohort, the median follow-up duration was 26 months [interquartile range (IQR) 16–38 months] overall. Specifically, the Castor group exhibited a median follow-up of 30 months (IQR 22–38 months), while the partial coverage group had a median follow-up of 24 months (IQR 14–36 months). The study encompassed a cumulative total of 433.8 person-years of follow-up, with 232.9 person-years attributed to the Castor group and 200.9 person-years to the partial coverage group.

### Adverse events at follow-up

Following PSMA, significant differences in perioperative outcomes were identified between the two groups. The median surgical time in the Castor group was 115 min [interquartile range (IQR), 100–145], which was significantly longer than the 80 min (IQR, 60–100) observed in the PartialCoverage group (*t* = −5.881, *p* < 0.01). In terms of perioperative adverse events, the incidence of cerebral infarction was significantly higher in the PartialCoverage group at 12.0% (11/92) compared to 3.2% (3/92) in the Castor group (*p* = 0.039). No statistically significant differences were observed between the two groups concerning postoperative true lumen diameter, postoperative in-hospital stay, pulmonary infection, left brachial artery aneurysm, puncture site infection, or renal failure (all *p* > 0.05). Furthermore, the all-cause mortality rates were similar between the two cohorts, with the Castor group exhibiting a rate of 6.5% (6 out of 92) and the PartialCoverage group showing a rate of 7.6% (7 out of 92), yielding a *p*-value of 1.000. The median follow-up periods were 30 months [interquartile range (IQR), 22–38 months] for the Castor group and 24 months (IQR, 14–36 months) for the PartialCoverage group, with the difference not reaching statistical significance (*p* = 0.077; see Table 2).

**Table 2:** Comparison of perioperative prognosis of after propensity matching.

Variables	Castor （*n* = 92)	PartialCoverage (*n* = 92)	*p*
Postoperative true lumen, mm	32 (30–34)	32 (30–34)	0.843
Surgical time, min	115 (100–145)	80 (60–100)	<0.001
Postoperative in-hospital stay, days	5 (4–7)	4 (3–6)	0.079
Pulmonary infection	4 (4.3)	3 (3.3)	1.000
Left brachial artery aneurysm	3 (3.3)	4 (4.3)	1.000
Puncture site infection	0	0	
Renal failure	0	0	
All-cause mortality	6 (6.5)	7 (7.6)	1.000
Follow-up time, months	30 (22–38)	24 (14–36)	0.077
Cardiovascular complication	0	0	
Aortic Rupture	1 (1.1)	0	
Recurrent aortic dissection or aneurysm	6 (6.5)	6 (6.5)	
Graft migration	0	0	
Endoleak	5 (5.4)	14 (15.2)	0.064
Cerebral infarction	3 (3.2)	11 (12)	0.039
Paraplegia	0	0	
Postoperative left subclavian artery occlusion	1 (1.1)	3 (3.3)	0.625

Data are shown in *n* (%), median (interquartile range; 25th–75th percentiles), *p*-value of <0.05 was considered statistically significant.

Kaplan–Meier survival curves for the two groups are depicted in Figures 1–3. Following PSMA, the cerebral infarction-free survival rate in the PartialCoverage group was significantly lower compared to the Castor group (HR = 0.228; 95% CI, 0.063–0.820; *p* = 0.013). This finding suggests that patients in the Castor group experienced a reduced risk of cerebral infarction during the follow-up period. Furthermore, no statistically significant differences were identified between the two groups concerning all-cause mortality or postoperative LSA occlusion-free survival after PSMA (both *p* > 0.05). The results indicate that the propensity score-matched analysis substantiates the superiority of the Castor strategy in mitigating the risk of cerebral infarction, whereas both approaches demonstrate equivalence concerning overall survival and LSA patency.

### Confounder adjustment, competing risk modeling, and relevant visual validation

Before adjustment, several covariates showed relatively high SMD values, indicating the presence of confounding bias between groups. After PSMA adjustment, the SMD values of all covariates were reduced and distributed within 0.2, indicating effective mitigation of confounding bias. Before adjustment, several covariates showed relatively high SMD values, indicating the presence of confounding bias between groups. After PSMA adjustment, the SMD values of all covariates were reduced and distributed within 0.2, indicating effective mitigation of confounding bias(Supplementary Figure 1). Multivariable Cox regression analysis: Using the Partial Coverage group as the reference, the Castor group had a hazard ratio (HR) for mortality of 0.55 (95% CI: 0.23–1.27, *p* = 0.160), which was not statistically significant(Supplementary Table 1). Using the Partial Coverage group as the reference, the Castor group was associated with a significantly reduced risk of cerebral infarction (HR = 0.30, 95% CI: 0.09–1.00, *p* = 0.049)(Supplementary Table 2). After accounting for Mortality as a competing event, results were highly consistent with those from Cox regression. The Castor group remained associated with a significantly reduced risk of cerebral infarction (HR = 0.31, 95% CI: 0.09–1.00, *p* = 0.049) (Supplementary Table 3). Comparison of baseline characteristics after IPTW adjustment (Partial Coverage group, *n* = 268; Castor group, *n* = 107). Between-group comparisons for all covariates, including demographic characteristics (age, sex), comorbidities (aortic calcification, coronary heart disease, hypertension, diabetes mellitus, uremia, previous cerebral infarction, respiratory disease, pleural effusion), and imaging parameters (distance from the intimal tear to the ostium of the left subclavian artery, preoperative true lumen/false lumen diameter), yielded *p*-values > 0.05 (range 0.151–0.921). These findings indicate no statistically significant between-group differences in baseline characteristics after IPTW adjustment, with effective balancing of confounding factors, providing a reliable foundation for subsequent prognostic analyses(Supplementary Table 4). Before adjustment, several covariates showed relatively high SMD values, indicating the presence of confounding bias between groups. After IPTW adjustment, the SMD values of all covariates were reduced and distributed within 0.1, confirming that IPTW effectively balanced confounding factors between groups and substantially reduced the risk of bias (Supplementary Figure 2). Kaplan–Meier survival curves for mortality after IPTW adjustment were plotted. The curves intuitively demonstrate the cumulative survival trends with respect to mortality in both groups during follow-up. The results showed no statistically significant difference between the Castor group and the Partial Coverage group, further validating the findings from multivariable Cox regression analysis. No significant statistical difference in the impact of the two treatment strategies on patient mortality risk was identified (Supplementary Figure 3). Kaplan–Meier survival curves for cerebral infarction after IPTW adjustment were plotted. The log-rank test showed *p* = 0.005, with a hazard ratio (HR) for cerebral infarction of 0.21 (95% CI: 0.07–0.68) between groups. These results further validate, from the perspective of survival trends, that the risk of cerebral infarction in the Castor group is lower than that in the Partial Coverage group, with a statistically significant between-group difference in survival (Supplementary Figure 4).

## Discussion

The present study demonstrated that Castor branched stent revascularization of the LSA was associated with a significantly lower incidence of postoperative cerebral infarction compared with partial coverage, while all-cause mortality and the cumulative incidence of postoperative LSA occlusion were comparable between the two surgical approaches.

Intraoperative metrics revealed that the operative time was significantly longer in the Castor group than in the partial LSA coverage group (115 min vs. 80 min, *p* < 0.01). This difference was attributed to the comparatively more complex operational process of Castor branched stent revascularization and its stricter requirements for precise positioning and stent deployment. Studies have shown that procedures involving branched stent reconstruction significantly increase time consumption due to precise positioning and complex endovascular operations (22). Nevertheless, no significant between-group differences were observed in the incidence of major perioperative complications (e.g., pulmonary infection, left brachial artery pseudoaneurysm), suggesting that the two surgical approaches are clinically comparable with regard to short-term safety. This finding aligns with the results of a number of published studies focusing on the perioperative safety of various LSA management techniques in TEVAR.

With regard to long-term prognosis, significant differences were observed between the two groups. Although all-cause mortality and the incidence of postoperative LSA occlusion did not differ significantly between the groups, the partial LSA coverage group had a significantly higher incidence of cerebral infarction compared with the Castor group (12% vs. 3.2%, *P* = 0.039). This discrepancy may be explained by hemodynamic alterations following partial LSA coverage that impair cerebral perfusion. Hemodynamic simulation analyses in previous studies have demonstrated that partial LSA coverage modifies aortic arch blood flow patterns, compromises cerebral perfusion via the vertebral and carotid artery systems, and increases the risk of cerebral infarction (23). Meanwhile, the PartialCoverage group also showed a trend toward a higher incidence of endoleak compared with the Castor group (15.2% vs. 5.4%, *p* = 0.064), although this difference did not reach statistical significance. The occurrence of endoleak may adversely affect short- and long-term surgical outcomes and elevate the risk of serious adverse events, such as aortic rupture or aneurysm expansion. Several studies have demonstrated that endoleak is one of the key factors influencing the long-term efficacy of TEVAR, with significant differences in endoleak incidence among different LSA management strategies (24, 25).

Notably, Kaplan–Meier survival curve analysis showed that cerebral infarction-free survival rates were similar between the two groups before PSMA, but the hazard of incident cerebral infarction in the Castor group was significantly lower than that in the partial LSA coverage group after matching. This indicates that Castor stent revascularization of the LSA confers a significant advantage in reducing the risk of postoperative cerebral infarction after adjusting for potential clinical confounders. However, the follow-up duration in the partial LSA coverage group was relatively shorter (24 months vs. 30 months, *P* = 0.077, approaching statistical significance), which may introduce bias into the evaluation of long-term prognosis. Longer-term follow-up data are warranted for further verification (18).

In recent decades, a prospective study (18) has explored the strategy of LSA reconstruction during thoracic endovascular aortic repair TEVAR. In a single-group prospective clinical trial conducted at 11 cardiovascular centers in East Asia, 73 patients with TBAD were enrolled. Technical failure occurred in 2 cases, with an endoleak rate of 5%, 1 case of in-hospital mortality, and no major complications. The median follow-up period was 61 months [interquartile range (IQR), 48–72 months], with a 1-year mortality rate of 5% and a 6-year mortality rate of 7%. Two new entry tears developed at the stent-graft margins and were successfully managed with endovascular repair; the patency rate of the reconstructed LSA segment during follow-up was 93%. These results are consistent with those of our study after PSMA. However, this single-group prospective design carries a high risk of bias and lacks a parallel control group, which limits the validity of its results. Although our study is a retrospective analysis, it includes patients from a more representative real-world clinical setting.

Recent retrospective studies (22, 26) have proposed partial LSA coverage as a feasible strategy for TBAD patients with a proximal landing zone <2 cm, with no 30-day adverse events (e.g., death, aortic rupture, paraplegia) reported. However, these findings were constrained by small cohorts and a short follow-up duration. Our study confirmed that partial LSA coverage did not increase short-term risks (all-cause mortality, cerebral infarction, endoleak), supporting its short-term safety. Long-term follow-up, however, revealed a higher incidence of cerebral infarction, which is consistent with prior research (23). This highlights the clinical importance of LSA revascularization—either immediate or delayed—as corroborated by other studies (27), to mitigate potential long-term neurological complications.

### Study limitations

This study also has limitations. First, it is a single-center retrospective comparative study. Although PSMA was used to reduce bias, it still cannot completely eliminate the influence of potential confounding factors. A randomized controlled trial (RCT) is a more ideal choice, but retrospective studies with PSMA can be accepted when an insufficient number of RCTs have been conducted. Second, the sample size is relatively limited, which may affect the statistical power of the results. For complications with low incidence, the insufficient sample size may make it impossible to accurately assess differences.

## Conclusions

In summary, following adjustment for baseline patient demographics and clinical characteristics between the two surgical cohorts via PSMA, mid-term outcomes indicated that TEVAR with LSA reconstruction using the Castor branched stent was associated with a lower incidence of cerebral infarction compared with partial LSA coverage.

## Data Availability

The data analyzed in this study is subject to the following licenses/restrictions: limitations of the dataset study design constraint: this is a single-center retrospective study. Although propensity score matching (PSMA) was used to balance baseline variables, it cannot fully eliminate the impact of potential confounding factors, resulting in a lower level of evidence compared to randomized controlled trials (RCTs). Insufficient Sample Size: The overall sample size (377 cases) and the matched sample size (92 cases per group) are relatively limited, which may reduce statistical power and hinder accurate assessment of differences in low-incidence complications between groups. Difference in follow-up duration: the follow-up duration of the partial left subclavian artery (LSA) coverage group (24 months) was slightly shorter than that of the Castor stent group (30 months), with a near-statistically significant difference (*P* = 0.077). This may introduce bias into the evaluation of long-term prognosis, and longer follow-up data are needed for further verification. Lack of external validation: data were only collected from a single center (The First Affiliated Hospital of Anhui Medical University), and no multicenter cohorts were included. Thus, the external generalizability of the study results requires further validation. Requests to access these datasets should be directed to Shenglin Ge email: geshenglin@ahmu.edu.cn.

## Supplementary material

The Supplementary Materialfor this article can be found online at: https://www.frontiersin.org/articles/10.3389/fcvm.2026.1777371/full#supplementary-material
