# Supplementary material for: Castor stent reconstruction of left subclavian artery reduces the incidence of postoperative cerebral infarction of type B aortic dissection with insufficient proximal landing area: a propensity score matched analysis
Source: Front Cardiovasc Med. 2026 Mar 12;13:1777371. doi: 10.3389/fcvm.2026.1777371 (PMC13017327; doi:10.3389/fcvm.2026.1777371)
Supplement: Supplementary file 1 [file Datasheet1.pdf]

APPENDIX A. SUPPLEMENTARY FIGURES AND TABLES

This retrospective study aimed to compare the prognostic differences between two treatment strategies (the Castor group and the Partial Coverage group). In response to the reviewer's request, we have supplemented analyses including confounder adjustment, competing risk modeling, and relevant visual validation. Propensity score matching (PSM) and inverse probability of treatment weighting (IPTW) were used to balance covariates between groups. Multivariable Cox regression, competing risk models, and Kaplan–Meier (KM) survival curves were applied for outcome analyses. The balancing performance was further verified using standardized mean difference (SMD) distribution plots and baseline characteristic tables. The detailed results are described as follows:

Supplementary Figure S1 Standardized mean difference (SMD) distribution of covariates before and after propensity score matching (PSMA)

Before adjustment, several covariates showed relatively high SMD values, indicating the presence of confounding bias between groups. After PSMA adjustment, the SMD values of all covariates were reduced and distributed within 0.2, indicating effective mitigation of confounding bias.

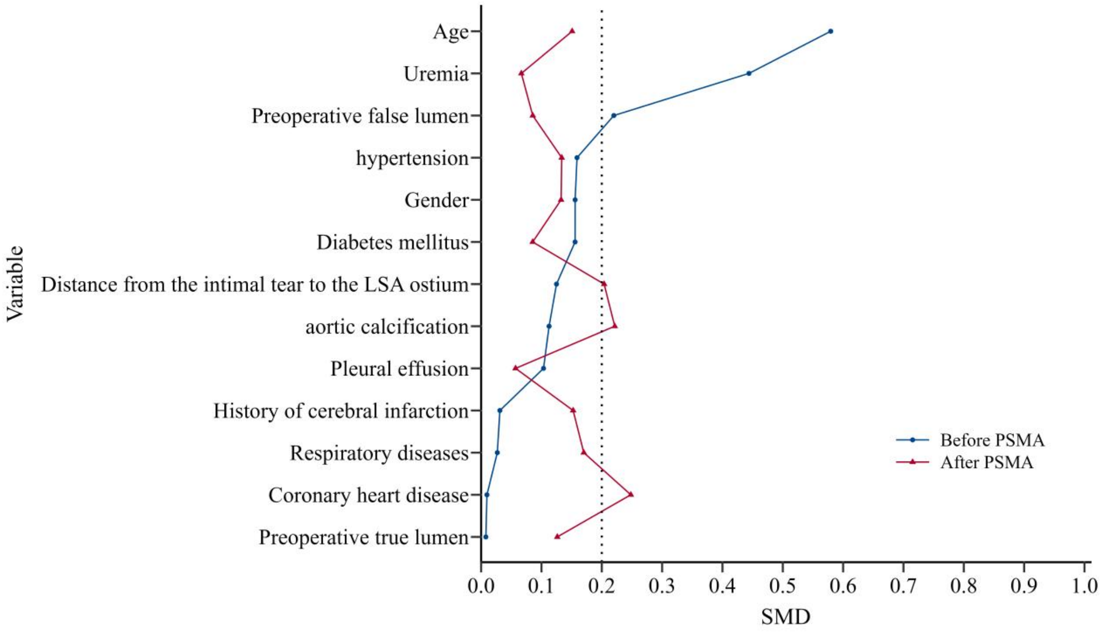

Supplementary Table S1 Multivariable Cox Regression Analysis for Mortality

Multivariable Cox regression analysis: Using the Partial Coverage group as the reference, the Castor group had a hazard ratio (HR) for mortality of 0.55 (95% CI: 0.23–1.27, p = 0.160), which was not statistically significant.

Variables	HR (95%CI)	p value
Group		
PartialCoverage	Ref.	
Castor	0.55 (0.23 to 1.27)	0.160
Age	1.02 (0.99 to 1.06)	0.164
Gender		
0	Ref.	
1	1.00 (0.44 to 2.25)	0.999

Variables	HR (95%CI)	p value
aortic_calcification		
0	Ref.	
1	0.62 (0.12 to 3.08)	0.557
Coronary_heart_disease		
0	Ref.	
1	1.77 (0.38 to 8.34)	0.470
hypertension		
0	Ref.	
1	1.31 (0.53 to 3.29)	0.559
Diabetes_mellitus		
0	Ref.	
1	1.54 (0.18 to 12.87)	0.689
Uremia		
0	Ref.	
1	1.95 (0.92 to 4.16)	0.084
History_of_cerebral_infarction		
0	Ref.	
1	1.61 (0.78 to 3.33)	0.202
Respiratory_diseases		
0	Ref.	
1	1.13 (0.20 to 6.45)	0.887
Pleural_effusion		
0	Ref.	
1	0.87 (0.31 to 2.51)	0.802
Distance_from_the_intimal_tear_to_the_LSA_ostium	0.99 (0.92 to 1.07)	0.869
Preoperative_true_lumen	1.05 (0.92 to 1.20)	0.458
Preoperative_false_lumen	1.00 (0.89 to 1.13)	0.962

Supplementary Table S2 Multivariable Cox Regression Analysis for Cerebral Infarction

Using the Partial Coverage group as the reference, the Castor group was associated with a significantly reduced risk of cerebral infarction (HR = 0.30, 95% CI: 0.09–1.00, p = 0.049).

Variables	HR (95%CI)	p value
Group		
PartialCoverage	Ref.	
Castor	0.30 (0.09 to 1.00)	0.049
Age	0.97 (0.93 to 1.01)	0.148
Gender		
0	Ref.	
1	0.98 (0.33 to 2.92)	0.972
aortic_calcification		
0	Ref.	
1	0.00 (0.00 to Inf)	0.996
Coronary_heart_disease		
0	Ref.	
1	4884046127.76 (0.00 to Inf)	0.996
hypertension		
0	Ref.	
1	3.18 (0.71 to 14.34)	0.132
Diabetes_mellitus		
0	Ref.	
1	3.53 (0.34 to 36.69)	0.291

Variables	HR (95%CI)	p value
Uremia		
0	Ref.	
1	1.48 (0.53 to 4.12)	0.457
History_of_cerebral_infarction		
0	Ref.	
1	3.03 (1.07 to 8.59)	0.036
Respiratory_diseases		
0	Ref.	
1	1.96 (0.32 to 11.91)	0.464
Pleural_effusion		
0	Ref.	
1	1.87 (0.61 to 5.71)	0.274
Distance_from_the_intimal_tear_to_the_LSA_ostium	0.98 (0.90 to 1.08)	0.728
Preoperative_true_lumen	0.89 (0.73 to 1.07)	0.214
Preoperative_false_lumen	0.94 (0.80 to 1.10)	0.434

Supplementary Table S3 Multivariable Competing Risk Regression Analysis for Cerebral Infarction

After accounting for Mortality as a competing event, results were highly consistent with those from Cox regression. The Castor group remained associated with a significantly reduced risk of cerebral infarction (HR = 0.31, 95% CI: 0.09–1.00, p = 0.049).

Variables	HR (95%CI)	p value
Group		
PartialCoverage	Ref.	
Castor	0.31 (0.09 to 1.00)	0.049
Age	0.97 (0.94 to 1.01)	0.121
Gender		
0	Ref.	
1	0.96 (0.30 to 3.15)	0.952
aortic_calcification		
0	Ref.	
1	0.06 (0.00 to 1.05)	0.054
Coronary_heart_disease		
0	Ref.	
1	17.42 (1.44 to 211.42)	0.025
hypertension		
0	Ref.	
1	2.94 (0.60 to 14.31)	0.181
Diabetes_mellitus		
0	Ref.	
1	4.19 (0.16 to 107.92)	0.387
Uremia		
0	Ref.	
1	1.74 (0.67 to 4.52)	0.253
History_of_cerebral_infarction		
0	Ref.	
1	2.59 (0.84 to 7.99)	0.097
Respiratory_diseases		
0	Ref.	
1	2.06 (0.34 to 12.65)	0.434
Pleural_effusion		

Variables	HR (95%CI)	p value
0	Ref.	
1	1.71 (0.55 to 5.31)	0.350
Distance_from_the_intimal_tear_to_the_LSA_ostium	1.00 (0.90 to 1.11)	0.985
Preoperative_true_lumen	0.85 (0.72 to 1.01)	0.068
Preoperative_false_lumen	0.92 (0.80 to 1.07)	0.291

Note: Mortality was treated as a competing event.

Supplementary Table S4 Baseline Characteristics After Inverse Probability of Treatment Weighting (IPTW)

Comparison of baseline characteristics after IPTW adjustment (Partial Coverage group, n=268; Castor group, n=107). Between-group comparisons for all covariates, including demographic characteristics (age, sex), comorbidities (aortic calcification, coronary heart disease, hypertension, diabetes mellitus, uremia, previous cerebral infarction, respiratory disease, pleural effusion), and imaging parameters (distance from the intimal tear to the ostium of the left subclavian artery, preoperative true lumen/false lumen diameter), yielded p-values > 0.05 (range 0.151–0.921). These findings indicate no statistically significant between-group differences in baseline characteristics after IPTW adjustment, with effective balancing of confounding factors, providing a reliable foundation for subsequent prognostic analyses.

Characteristic	PartialCoverage N = 268	Castor N = 107	p-value
Age	60.00 (51.00, 71.00)	59.00 (48.00, 74.00)	0.674
Gender			0.818
0	59 (22)	22 (21)	
1	209 (78)	85 (79)	
aortic_calcification			0.639
0	197 (74)	76 (71)	
1	70 (26)	31 (29)	
Coronary_heart_disease			0.484
0	207 (77)	79 (73)	
1	60 (23)	29 (27)	
hypertension			0.855
0	49 (18)	21 (19)	
1	218 (82)	86 (81)	
Diabetes_mellitus			0.747
0	264 (99)	105 (98)	
1	4 (1.4)	2 (1.8)	
Uremia			0.855
0	195 (73)	77 (72)	
1	73 (27)	30 (28)	
History_of_cerebral_infarction			0.699
0	200 (75)	82 (77)	
1	68 (25)	25 (23)	
Respiratory_diseases			0.701
0	258 (96)	104 (97)	
1	10 (3.7)	3 (3.0)	
Pleural_effusion			0.921
0	224 (84)	90 (84)	
1	43 (16)	17 (16)	
Distance_from_the_intimal_tear_to	6.00 (2.00, 10.00)	6.00 (2.00, 10.00)	0.621

Characteristic	PartialCoverage<br>N = 268	Castor<br>N = 107	p-value
_the_LSA_ostium			
Preoperative_true_lumen	11.00 (9.00, 12.00)	12.00 (10.00, 14.00)	0.151
Preoperative_false_lumen	18.00 (17.00, 22.00)	19.00 (16.00, 22.00)	0.575

Supplementary Figure S2 Standardized mean difference (SMD) distribution of covariates before and after IPTW

Before adjustment, several covariates showed relatively high SMD values, indicating the presence of confounding bias between groups. After IPTW adjustment, the SMD values of all covariates were reduced and distributed within 0.1, confirming that IPTW effectively balanced confounding factors between groups and substantially reduced the risk of bias.

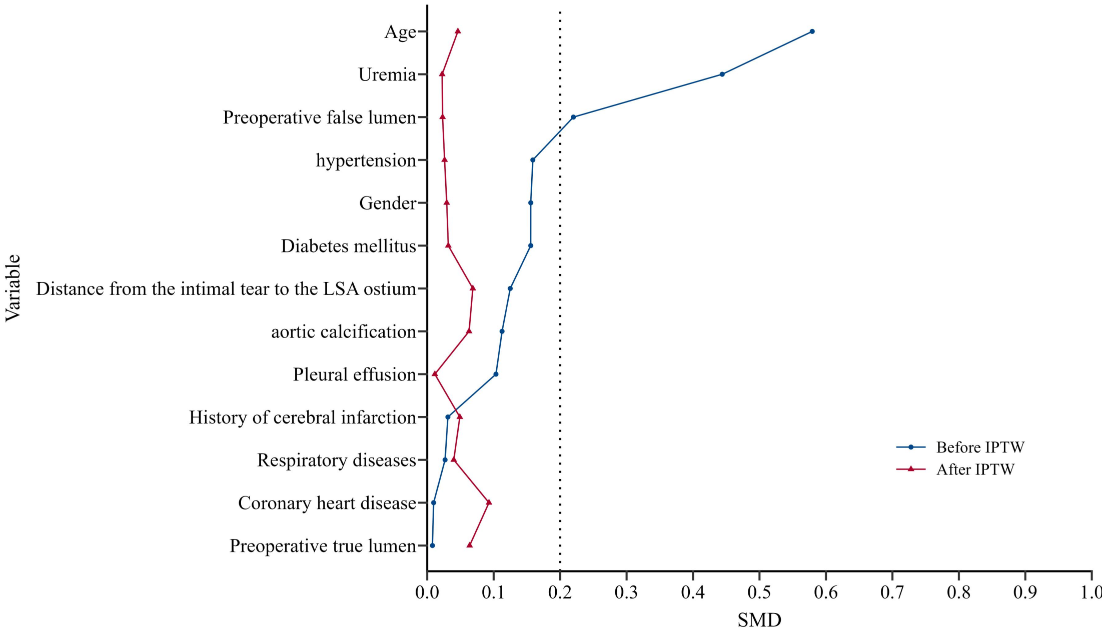

Supplementary Figure S3 Kaplan–Meier curves Analysis of Mortality After IPTW Adjustment

Kaplan–Meier survival curves for mortality after IPTW adjustment were plotted. The curves intuitively demonstrate the cumulative survival trends with respect to mortality in both groups during follow-up. The results showed no statistically significant difference between the Castor group and the Partial Coverage group, further validating the findings from multivariable Cox regression analysis. No significant statistical difference in the impact of the two treatment strategies on patient mortality risk was identified.

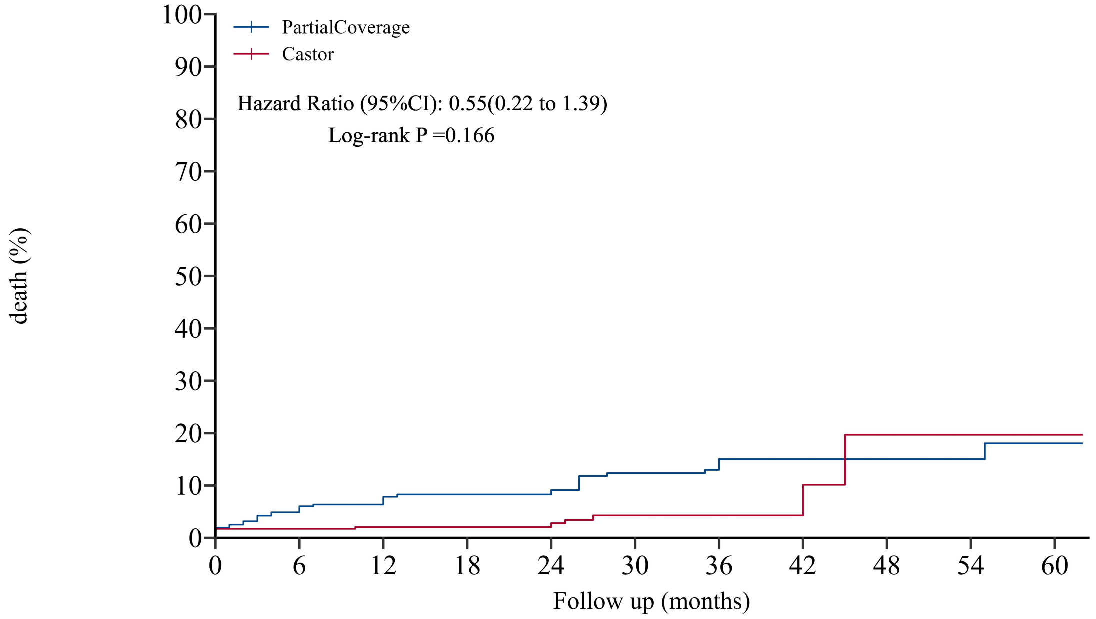

Number at risk (number event)

PartialCoverage	268 (0)	249 (16)	241 (5)	206 (1)	170 (2)	123 (5)	110 (3)	77 (0)	57 (0)	23 (0)	6 (1)
Castor	107 (0)	105 (2)	103 (0)	89 (0)	79 (1)	60 (1)	40 (0)	23 (1)	14 (2)	6 (0)	4 (0)

Supplementary Figure S4 Kaplan–Meier curves Analysis of Cerebral Infarction After IPTW Adjustment

Kaplan–Meier survival curves for cerebral infarction after IPTW adjustment were plotted. The log-rank test showed p = 0.005, with a hazard ratio (HR) for cerebral infarction of 0.21 (95% CI: 0.07–0.68) between groups. These results further validate, from the perspective of survival trends, that the risk of cerebral infarction in the Castor group is lower than that in the Partial Coverage group, with a statistically significant between-group difference in survival.

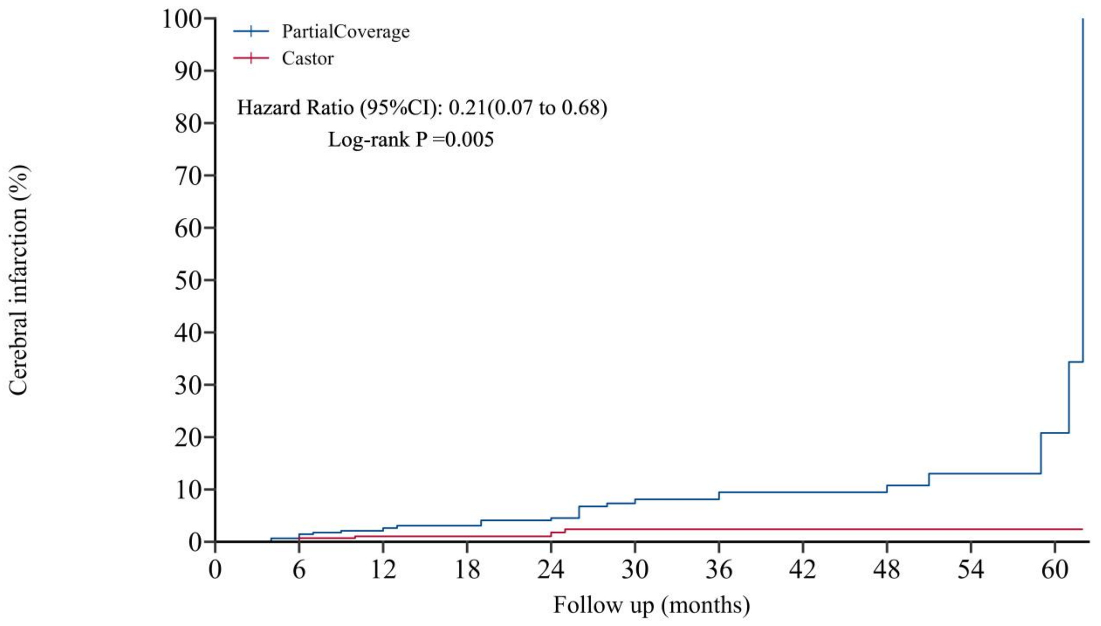

Number at risk (number event)

	0	6	12	18	24	30	36	42	48	54	60
PartialCoverage	268 (0)	249 (4)	241 (3)	206 (1)	170 (3)	123 (5)	110 (2)	77 (0)	57 (1)	23 (1)	6 (1)
Castor	107 (0)	105 (1)	103 (0)	89 (0)	79 (1)	60 (0)	40 (0)	23 (0)	14 (0)	6 (0)	4 (0)

Supplementary Table S5

Comparison of perioperative prognosis of after propensity matching

	Castor （n = 92）	PartialCoverage （n = 92）	*p*
Left vertebral artery dominance	4(4.3%)	3(3.3%)	1.000
Ostial stenosis of the LSA	3(3.3%)	4(4.3%)	1.000
Carotid artery atherosclerotic plaque formation	1（1.1%）	0	

Data are shown in n (%), median (interquartile range; 25th–75th percentiles), *p*-value of < 0.05 was considered statistically significant.
